# Stevia vs. Sucrose: Influence on the Phytochemical Content of a Citrus–Maqui Beverage—A Shelf Life Study

**DOI:** 10.3390/foods9020219

**Published:** 2020-02-19

**Authors:** Francisco J. Salar, Vicente Agulló, Cristina García-Viguera, Raúl Domínguez-Perles

**Affiliations:** Phytochemistry and Healthy Foods Lab. Group of Quality, Safety, and Bioactivity of Plant Foods. Department of Food Science and Technology, (CEBAS-CSIC), University Campus of Espinardo, 30100 Murcia, Spain

**Keywords:** sweeteners, anthocyanin, flavanones, vitamin C, processing

## Abstract

The consumption of sugar-sweetened beverages has been related with the risk of cardiovascular diseases and other pathophysiological situations, such as obesity or diabetes mellitus. Given the increasing awareness on this fact, food industries are developing new products to reduce the amount of added sugar in development of food products development. Accordingly, in the present work, new functional beverages, constituting a dietary source of bioactive phenolics and supplemented with stevia or sucrose, were designed in order to study the influence of the sweetener during processing and shelf-life. This study is of critical for the informed selection of the sweetener based on its effect on the final phytochemical profile of beverages, especially taking into consideration that there are no previous studies on *Stevia rebaudiana*. Physicochemical features and phytochemical composition, as well as stability of the different beverages concerning these parameters, were evaluated for 90 days during storage under different conditions (refrigeration (4 °C) and room temperature (25 °C) under light or darkness conditions). Physicochemical parameters (pH, titratable acidity, total soluble solids, and color) did not display statistically significant differences between beverages. Storage temperature was the greatest determinant affecting the stability of all the analyzed bioactive compounds (vitamin C, anthocyanins, and flavanones). The main difference between sweeteners was observed in flavanones, which exhibited a higher loss during storage under day light conditions when stevia was added instead of sucrose. In addition, the juices’ colors were rather stable, keeping a reddish coloration and natural appearance throughout the shelf life. Hence, stevia could be considered as an alternative sweetener by the beverage industry.

## 1. Introduction

Cardiovascular diseases (CVDs) are the main cause of human death, responsible for up to 17.9 million deaths per year (31.0% of global deaths) (http://www.who.int, 2019). A significant proportion of CVDs are consequences of inappropriate dietary habits that influence other physiopathological conditions (obesity, diabetes mellitus, and high cholesterol, among others) closely linked to the incidence of CVDs [1]. Among the factors contributing to the severity of CVDs and comorbidities, epidemiological studies have identified the relationship with the consumption of sugar-sweetened beverages [2,3].

Prompted by epidemiological evidence, food and beverage industries are currently developing products with lower natural sugar (sucrose or fructose) content, focusing their activity on the development of new formulations, using diverse types of water, juices, and nonalcoholic drinks which preserve nutrients and reduce the glycemic content. In the frame of this trend, industries base their strategy on the addition of other sweeteners, which could constitute an alternative to decrease the intake of added sugars and thus counteract the postprandial response [4,5]. Nevertheless, a controversy regarding the use of artificial sweeteners exists because of their effect on the caloric intake and obesity [6] as well as a growing interest in natural sweeteners (stevia—*Stevia rebaudiana*—for instance) instead of artificial ones. In this aspect, stevia is of special interest, due to the healthy attributes reported in the scientific literature for this sweetener, such as antibacterial, diuretic, anti-inflammatory, anticarcinogenic, and antioxidant properties as well as their functionality as hyperglycemic and hypertensive inhibitors, among others [7,8]. In addition, recent studies have suggested stevia as protective for diverse phytochemical compounds, such as anthocyanins or vitamin C in model systems [9], although the scientific literature regarding interactions between stevia and other food components is still scarce and this issue merits to be further explored.

For developing new healthy foods and beverages using nonsugar sweeteners, fruits with high content of bioavailable and bioactive compounds are selected [10,11,12] such as red fruits (berries) rich in antioxidants and other bioactive compounds, particularly anthocyanins and other uncolored phenolics [13], as well as additional bioactive nutrients [14]. In respect to the health-promoting features of these fruits, during the last several years maqui berry (*Aristotelia chilensis* (Mol.) Stunz), a common edible berry native from southern Chile and part of neighboring Argentina, has emerged as a product of special relevance as a food ingredient [15]. Actually, the nutritional and phytochemical characterization of this fruit, as well as its biological properties, have suggested the capacity of maqui anthocyanins to modulate hyperglycemia and insulin sensitivity [4] as well as to inhibit mechanisms involved in the absorption of sugars [5]. Moreover, other phenolic compounds present in maqui are also responsible for the high antioxidant capacity and the ability to inhibit adipogenesis and inflammation [16,17], thus decreasing oxidative stress and providing cardioprotective and neuroprotective effects [18,19,20].

On the other hand, citrus fruits provide bioactive compounds (e.g., flavonoids, mainly flavanones, hydroxycinnamic and benzoic acids, 1-feruloyl-β-d-glucopyranoside, and 1-sinapoyl-β-d-glucopyranoside), in addition to vitamins, minerals, dietary fiber, essential oils, and carotenoids, with a biological activity against pro-oxidant agents, cancer, cardiovascular disease, diabetes, and obesity [21]. The combination of citrus fruits with maqui berry juices gives rise to an acidic status that stabilizes color and polyphenolic composition, constituting a combination that needs to be further explored [22].

Given these antecedents, the present work covers the alternative use of stevia (natural noncaloric sweetener), instead of sucrose (caloric sweetener control), in the design of new beverages, formulated with citrus and maqui berries, in relation to processing behavior. Juices were assessed on their physical (color) and chemical (pH, titratable acidity (TA), and total soluble solids (TSS)) features, as well as on their phytochemical composition (individual and total phenolics, vitamin C). Stability of these parameters was monitored during 90 days of storage at 4 °C, and at 25 °C under daylight or darkness conditions.

## 2. Material and Methods

### 2.1. Chemicals and Reagents

The standard of the phenolic compound cyanidin 3-*O*-glucoside chloride was obtained from TransMIT (Geiben, Germany). Caffeic, gallic, and ellagic acids were from Sigma Aldrich (St.Louis, MO, USA), and quercetin-3-*O*-rutinoside and hesperidin were purchased from Merck (Darmstadt, Germany). Formic acid, methanol, acetonitrile, and ethylenediaminetetraacetic acid disodium salt 2-hydrate (EDTA) were all obtained from Panreac (Barcelona, Spain). L-Ascorbic (AA) and dehydroascorbic (DHAA) acids were obtained from Acros Organics (Morris, NJ, USA) and Sigma-Aldrich (St. Louis, MO, USA), respectively. All solutions were prepared with ultrapure water from a Milli-Q Advantage A10 ultrapure water purification system (Millipore, Burlington, MA, USA).

### 2.2. Fruits and Sweeteners

Fresh organic dry maqui powder was provided by Maqui New Life S.A. (Santiago, Chile). Cítricos de Murcia S.L. (Ceutí, Murcia, Spain) and AMC Grupo Alimentación Fresco y Zumos S.A. (Espinardo, Murcia, Spain) provided the citrus juices. Sucrose and stevia were purchased at AB Azucarera Iberia S.L. (Madrid, Spain) and Agriestevia S.L (Molina de Segura, Murcia, Spain), respectively.

### 2.3. Experimental Design

Maqui powder was mixed with citrus juices (lemon juice mixed with other citrus in a certain proportion, in order to reduce acidity and obtain a more acceptable beverage) to obtain the base drink [23,24]. After, sweeteners were added in different proportions, stevia 4 mg per 100 mL and sucrose 7.5 g per 100 mL, in order to give the same sweet acceptance (tested by an informal consumer panel). Drinks (400 L each) were pasteurized according to common industrial standards (85 °C for 15 s) for beverage composition and pH at the Universidad Miguel Hernández (Orihuela, Alicante, Spain) and bottled. Afterwards, a total of 18 bottles (330 mL each) were selected for each sweetener. In order to study the influence of light and temperature, ten aliquots of each bottle were stored in transparent plastic vials (56 mm 18 mm Ø; volume 10 mL) with plastic screw-caps, and stored at 4 °C and at 25 °C under light (daylight cycle—16 h photoperiod) or darkness conditions (24 h dark) for 90 days. Samples were labeled according to Table 1. All juices designed and experimental condition tested were prepared in triplicates (*n* = 3) and all analytical determinations were performed in triplicates (*n* = 3). The analyses were carried out every 15 days within the first 60 days of storage, and the last analyses were carried out at the end of storage period at day 90.

### 2.4. pH, Titratable Acidity, and Total Soluble Solids

Physicochemical parameters were evaluated as quality indexes. The pH values were measured using a pH-meter (GLP 21; Crison Ltd, Barcelona, Spain). The TA was determined by titrating 2 g of juice (rising 30 g of with Milli-Q water) with 0.1 mol/L NaOH (pH 8.1). Results were expressed as grams of citric acid per 100 mL of sample (g CA/100 mL). The TSS content of the samples was recorded in a refractometer (Abbe WYA-S, Optic Ivymen® System; Biotech SL, Barcelona, Spain) at 20 °C with values being expressed as °Brix.

### 2.5. Qualitative and Quantitative Analysis of Phenolic Compounds

The identification and quantification of phenolic compounds was performed by applying the method previously reported in the literature [11,22], with a few modifications. Samples (2 mL) were centrifuged for 5 min at 21,382 rcf (model Sigma 1–13, B. Braun Biotech International, Osterode, Germany). Supernatants were filtered through a 0.45 mm PVDF filter (Millex HV13, Millipore, Burlington, MA, USA) and analyzed by HPLC-DAD-ESI/MSn and RP-HPLC-DAD for the identification and quantification, respectively. HPLC-DAD-ESI/MSn analyses were performed using an Agilent HPLC 1100 series equipped with a photodiode array detector and a mass detector in series (Agilent Technologies, Waldbronn, Germany). For identification of phenolic compounds, mass spectrometry data were acquired in the positive and negative ionization modes for anthocyanins and other phenolic compounds, respectively.

The phenolic compounds from samples (20 µL) were qualified and quantified in a chromatographic system with a Luna 5µm C18, 100 Å column (250 × 4.6 mm) using Security Guard Cartridges PFD C18 4 × 3.0 mm, both supplied by Phenomenex (Torrens, CA, USA). Chromatographic separation was performed using 5% formic acid (solvent A) and methanol (solvent B), using a linear gradient (time, %B) (0, 15%); (20, 30%); (30, 40%); (35, 60%); (40, 90%); (44, 90%), and back to initial conditions, allowing 10 min for column stabilization. The quantification was carried out using an Agilent Technologies 1220 Infinity Liquid Chromatograph equipped with an autoinjector (G1313, Agilent Technologies, Santa Clara, CA, USA) and a Diode Array Detector (1260, Agilent Technologies, Santa Clara, CA, USA). The flow rate was 0.9 mL/min. Chromatograms were recorded at 280, 320, 360, and 520 nm and processed on an Agilent ChemStation for LC 3D systems. The diverse phenolic compounds were identified by comparison with authentic standards of analytical grade. Flavanones were quantified as hesperidin at 280 nm, caffeic acid derivatives as caffeic acid at 320 nm, flavonols and ellagic acid as quercetin 3-*O*-rutinoside and ellagic acid, respectively, both at 360 nm, and anthocyanins as cyanidin 3-*O*-glucoside at 520 nm. The concentration of phenolic compounds was expressed as mg per 100 mL of juice.

### 2.6. Extraction and Analysis of Vitamin C

The content of vitamin C was determined by applying the UHPLC-ESI-QqQ-MS/MS-based method recently developed [25] and calculated by comparison with freshly prepared AA and DHAA authentic standards curves. The results were expressed as mg per 100 mL of juice.

### 2.7. Color Measurements

The color measurement was developed using the Konica Minolta Chroma Meter CR-5 supplied by Konica Minolta, Inc. (Osaka, Japan). Color parameters determined were luminosity (CIEL*), redness (a*), and yellowness (b*). Hue angle (*H*), Chroma (C), and ΔE were calculated from tan^−1^ (b*/a*), (a*^2^ + b*^2^)^1/2^, and (da*^2^ + db*^2^ + dL*^2^)^1/2^, respectively.

### 2.8. Statistical Analyses

Results are presented as means ± SD (*n* = 3). A paired *t*-test was developed to compare two parameters, and an analysis of variance (ANOVA) and Tukey’s multiple range tests were carried out to compare three or more conditions. All statistical analyses were performed using SPSS 19.0 software (LEAD Technologies, Inc., Chicago, IL, USA). The level of statistical significance was set at *p* < 0.05.

## 3. Results and Discussion

### 3.1. Quality Parameters

Results indicated that pH and titratable acidity were similar in all beverages (Table 2). Regarding TSS, there were significant differences between sucrose and stevia, as these contents were higher in those juices with added sucrose [26]. Nevertheless, initial values did not change during storage under the three studied conditions. This fact could be attributed to an outstanding stability of sucrose during heating, under both low and neutral pH conditions, and to the chemical degradation of stevia under extreme conditions of high temperature and pH [27].

### 3.2. Vitamin C

**Figure 1:**
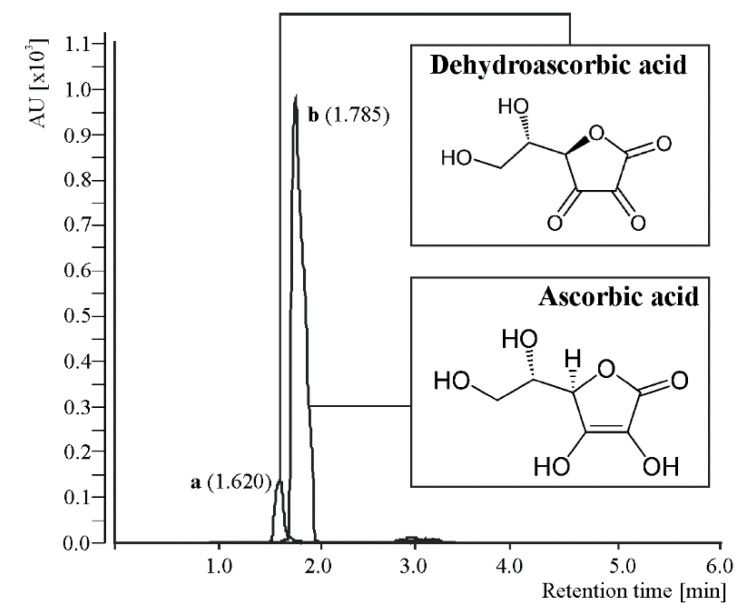
Representative overlap spectra of dehydroascorbic acid (**a**) and ascorbic acid (**b**) obtained by UHPLC-ESI-QqQ-MS/MS working in the multiple reaction monitoring mode.

The amount of vitamin C was provided uniquely by citrus juices, which are natural sources of this antioxidant, as it was not detected in maqui powder according to the quantification by UHPLC-ESI-QqQ-MS/MS (Figure 1).

**Figure 2:**
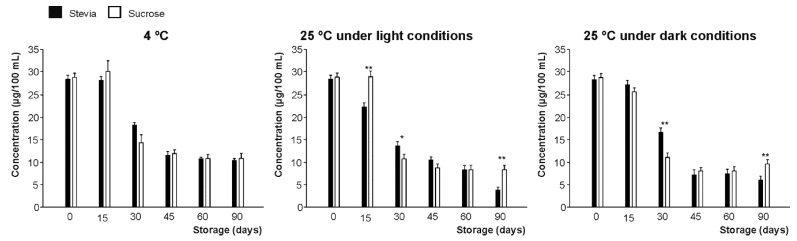
Vitamin C content (µg/100 mL) of juices developed using stevia and sucrose as sweeteners, measured during 90 days of storage under three different conditions (4 °C and 25 °C under light conditions and 25 °C under darkness conditions). Bars with asterisk(s) are significantly different according to the paired *t*-test developed at *p* < 0.05 (*) and *p* < 0.01 (**).

Initially, vitamin C content (calculated as the sum of AA and DHAA) was not significantly (*p* > 0.05) different (29 mg per 100 mL, on average) for the beverages prepared with the different sweeteners. However, even if the concentration of this vitamin at 4 °C presented non statistically significant differences during the first 15 days, these losses were significant during the second fortnight of the storage period (43.0% on average at 30 days). Finally, a significant decrease in both sweetener-type beverages (by 62% on average) throughout 90 days of storage (Figure 2) was observed, perhaps due to the mutual degradation between anthocyanins and vitamin C [28,29,30] under room temperature conditions, as discussed below.

In addition, storage temperature was identified as the most determining factor affecting the stability of vitamin C, in agreement with previous authors [31,32], while the presence or absence of light seems not to be critical, since it had a similar behavior under darkness and light conditions at 25 °C with a 70% loss on average.

### 3.3. Phenolic Composition of Juices

#### 3.3.1. Flavanones

**Figure 3:**
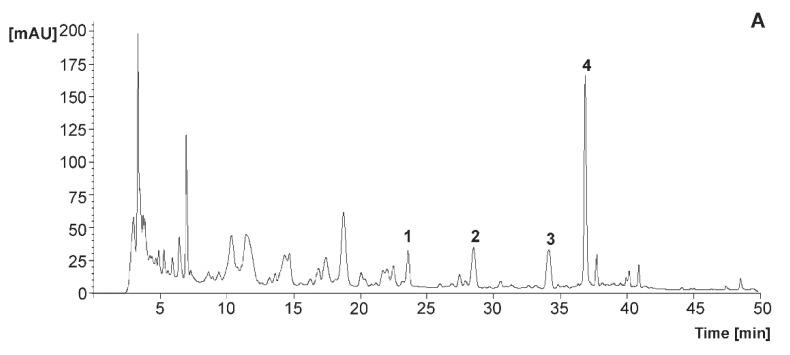

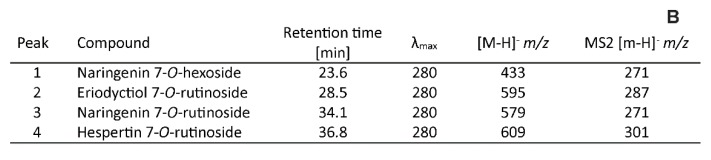
Representative chromatogram of the maqui–citrus juices recorded at 280 nm (**A**) and identity of the flavanones identified (**B**).

**Figure 4:**
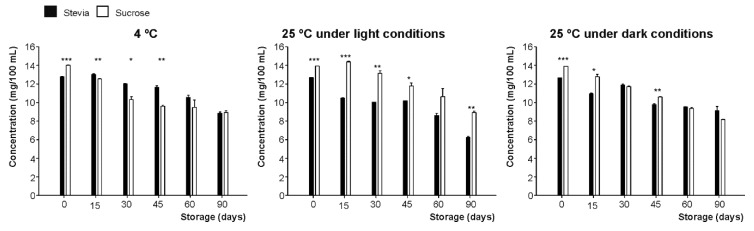
Content of total flavanones (mg/100 mL) of juices developed using stevia and sucrose as sweeteners, measured during storage for 90 days under three different conditions (4 °C and 25 °C under light conditions and 25 °C under darkness conditions). Bars with asterisk(s) are significantly different according to the paired *t*-test developed at *p* < 0.05 (*), *p* < 0.01 (**) and *p* < 0.001 (***).

When characterizing the phenolic composition of the juices elaborated in the frame of the present work, it was observed that, although the content of flavanones of fruit juices is closely dependent on the agro-environmental conditions associated with the fruits’ production [22,33], technological issues also should be considered as critical factors affecting the phenolic composition of the beverages [28]. Hence, regarding the drinks evaluated here, the flavanones were provided by citrus juices, with the most abundant being eriocitrin (eriodyctiol 7-*O*-rutinoside), narirutin (naringenin 7-*O*-rutinoside), and hesperidin (hesperetin 7-*O*-rutinoside) (Figure 3) with average initial concentrations of 1.69 mg/100 mL, 1.72 mg/100 mL and 9.98 mg/100 mL, respectively. The initial contents of total flavanones concentrations were 14.01 mg/100 mL and 12.78 mg/100 mL for sucrose and stevia, respectively (Figure 4).

Concerning variations on total flavanones content during storage, both juices (sucrose or stevia added) presented a significant total flavanones reduction, in agreement with previous studies [29]. These flavanone concentrations remained constant during the first 15 days of storage at 4 °C. After this initial period, the concentrations decreased in all juices, with a slightly higher concentration for those beverages with stevia added, until day 90, when a final 33% loss, on average, for both sweeteners was measured (Figure 4).

In contrast, in those drinks stored at 25 °C under light conditions, a higher progressive loss was observed for those with stevia added, reaching a final loss of 50% when compared to sugared ones (36% final loss) at the end of storage period. However, when stored under darkness conditions, the behavior was similar for both sweeteners as under refrigeration conditions.

On the other hand, taking into account the contribution of individual flavanones to the total concentration of this family of phenolics, both eriocitrin and narirutin did not display significant losses during the storage period, whereas hesperidin was the most affected by both sweeteners. Hence, the final losses, regarding the total content of flavanones, were mainly due to the degradation of hesperidin for all sweeteners and storage conditions. Thus, the decrease of hesperidin during storage found in the present work was in agreement with previous descriptions in the literature [22,30]. The available information in the literature about the degradation mechanism of flavanones during storage using stevia or other noncaloric sweeteners is scarce. However, according to the findings described in the present work, this is a central question that merits attention and further evaluation.

#### 3.3.2. Caffeic and Ellagic Acids

In addition to flavanones, phenolic acids were also present in the citrus–maqui beverages. In this respect, citrus juice was mainly responsible for the high amount of caffeic acid derivatives (5.30 mg/100mL, on average), which remained almost constant during 90 days of storage under all conditions.

On the other hand, free ellagic acid was also identified in the two beverages, exhibiting initial concentrations of 0.84 mg/100 mL, but in this case, provided by maqui berry, in accordance to previous phenolic characterizations of this fruit [16]. For both formulations, storage conditions caused modifications (decreases or augmentations) of its content along the storage period due to the degradation of ellagitanins to free ellagic acid [34]. In this sense, stevia and/or sucrose could interfere with the stability of ellagitanins and ellagic acid in a similar way.

#### 3.3.3. Anthocyanins

**Figure 5:**
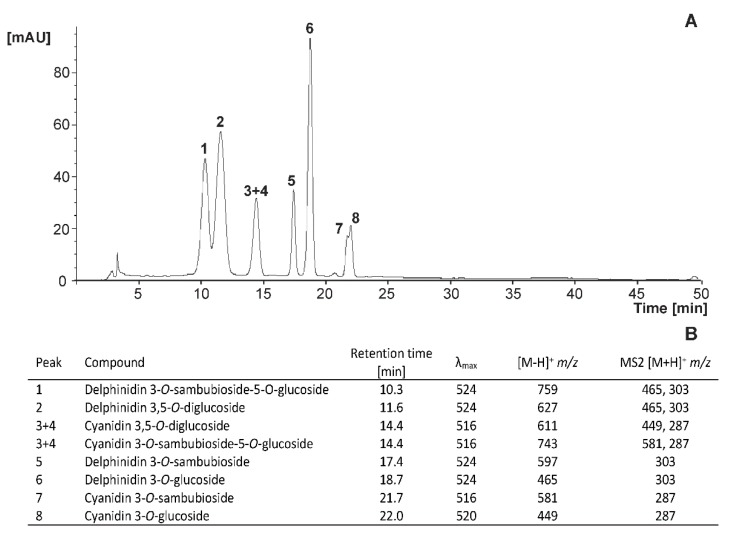
Representative chromatogram of the maqui-citrus juices recorded at 520 nm (**A**) and identity of the anthocyanins identified (**B**).

**Figure 6:**
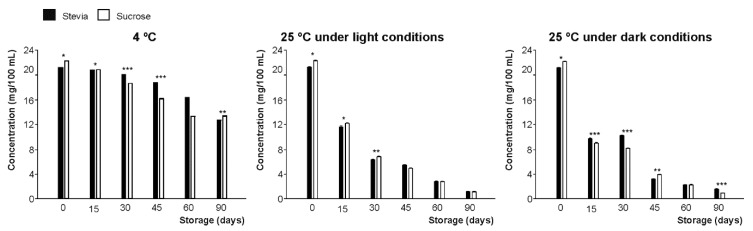
Content of total anthocyanins (µg/100 mL) of juices developed using stevia and sucrose as sweeteners, measured during storage for 90 days under three different conditions (4 °C and 25 °C under light conditions and 25 °C under dark conditions). Bars with asterisk(s) are significantly different according to the paired *t*-test developed at *p* < 0.05 (*), *p* < 0.01 (**), and *p* < 0.001 (***).

The range of anthocyanins present in *A. chilensis*, which has been extensively explored and described by Gironés et al. [35], were characterized in the new formulated beverages (Figure 5A,B). In this case, the relative abundance of total anthocyanins was similar for beverages prepared with both sweeteners (21.18 mg/100 mL for stevia and 22.20 mg/100 mL for sucrose), and the rate of anthocyanin degradation was gradual, reaching 40% loss after 90 days when stored at 4 °C (Figure 6).

Those samples stored at room temperature (under light or dark conditions) presented a higher decrease in their colored flavonoids, being reduced by 95%, on average, after 90 days. This finding was in agreement with previous descriptions that reported a statistically significant (*p* < 0.05) negative relationship between the storage temperature and the degradation of these compounds [36].

These diverse degradation rates of anthocyanins in the range of juices and storage conditions might be attributed to presence of vitamin C, supplied by the citrus fruits, accordingly to the negative contribution of vitamin C to the stability of anthocyanins [28,37]. In respect to this, two main mechanisms responsible for their mutual degradation have been reported. These mechanisms could first involve direct condensation reactions between the carbon 4 of anthocyanins and ascorbic acid [38] that cause the loss of both bioactive compounds, and second, the auto-oxidation of ascorbic acid as a consequence of the release of free radicals mechanism; these cleave the pyrilium ring of anthocyanins causing a loss of color [37,39,40,41]. Nevertheless, more research has to be done, as the results are inconsistent in the literature, depending on the anthocyanin and source of ascorbic acid.

### 3.4. Color Changes during Storage

The reddish coloration of the new beverage of maqui–citrus blend is attributed to the total content of anthocyanins. In this aspect, it has been reported that not only the concentration of total anthocyanins but also the content of ascorbic acid and temperature are key factors influencing the stability of the anthocyanins [38] and hence the color parameters of beverages.

During the 90 day of shelf life, lightness (CIEL* value) tended to increase not only for both sweeteners but also for all storage conditions (Table 3, Table 4 and Table 5). This behavior is in close agreement with previous findings described in the literature [22].

According to this, the evaluation of the color in these citrus–maqui based beverages presented a trend regarding a tentative correlation between CIEL* values and total anthocyanin content, which was even more evident in samples stored at 25 °C. This fact suggests that the anthocyanin degradation was related with the increased lightness over the storage period.

Moreover, as a general trend, a decrease in redness (CIEa*) was observed in all juices at different storage conditions. This occurred to a higher extent in those samples stored at 25 °C, which is in line with the total anthocyanin content degradation of the beverages. Nevertheless, this trend changed during the last 30 days of storage at 4 °C for all juices, probably due to the formation of colored polymers or copigmentation between anthocyanins and other flavonols that considerably increased visual color. This situation could mask adverse changes occurring through shelf life regarding the anthocyanin content of beverages [22,42]. On the other hand, only minor (not significant) variations were observed in respect to yellowness (CIEb* value) over time for the diverse beverages. In this aspect, changes of yellowness were represented by small increases during the 90-day storage and emphasized through the last 30 days for all the samples and storage conditions. With respect to chroma and hue angle, an increase was noticed for both sweeteners and storage conditions during the period monitored, indicating a numerical browning tendency, but minimally detected by the human eye.

Finally, color differences, measured by ΔE, changed during storage, which indicates differences in beverages [43]. In general, no visual alterations were noted during the first 2 months of storage, and only after 3 months were some color changes detected visually. In respect to the influence of storage temperature, at 4 °C color was better preserved until day 60, relative to those juices stored at 25 °C. Furthermore, the use of stevia or sucrose as sweeteners contributed to color alteration in a similar way, with slight differences. It has been established that differences in ΔE over 3.0 CIEL*a*b units could be the limit of an estimation of consumers acceptance for wine samples [44], however, these differences were not visual in our beverages, as the differences were only detected when surpassing 20 units.

It is important to highlight that, even if there was a decrease in the anthocyanin content over storage, the attractive reddish coloration of the drinks remained substantially stable during the 90 days monitored. Hence, the almost invariable color of the beverages during 3 months reinforces the theory that states that final color, during storage, could be a result of the formation of other colored polymers [33,45]. This could be due to metal complexation or intra- and intermolecular copigmentation, among other mechanisms [46]. Also, the newly formed molecules could provide brighter and more stable color throughout storage.

In addition, another factor that might contribute to the color achieved at the end of storage is the ascorbic acid content of the beverages. In this regard, the compounds resulting from the oxidative degradation of ascorbic acid, such as 2,3-diketogulonic acid, could contribute to browning reactions and thus to darkening the final color of the beverages [38].

## 4. Conclusions

The new designed beverages, based on maqui berry and citrus juices, have shown similar stability, regardless the sweetener added, of all the analyzed quality parameters, indicating that they could have promising health-promoting effects according to their content in bioactive vitamin C and phenolic compounds. Nevertheless, in the present work, storage temperature has been demonstrated as the determining parameter for the degradation of all the studied bioactive compounds, while light exposure does not seem to be a critical factor. Moreover, the color was rather stable, keeping a reddish coloration and natural appearance throughout the shelf life, although refrigeration conditions better preserved the color during storage.

Finally, the results obtained indicate that stevia could be considered as an alternative sweetener by the industry, even taking into consideration some differences noticed in comparison with sucrose, regarding a higher loss of the total flavanones content when stored under light conditions at room temperature and a slightly higher loss of vitamin C during the first month, under equal conditions.

Accordingly, storage of the newly developed beverages should be done under refrigeration conditions, and some specific studies should be performed to fine-tune the optimal preservation conditions for each sweetener.

## Figures and Tables

**Table 1:** Codification of samples included in the experimental design.

Code	Beverage and Storage Condition
ST4	Beverage with stevia stored at 4 °C under darkness conditions
ST25L	Beverage with stevia stored at 25 °C under light conditions
ST25O	Beverage with stevia stored at 25 °C under darkness conditions
SA4	Beverage with sucrose stored at 4 °C under darkness conditions
SA25L	Beverage with sucrose stored at 25 °C under light conditions
SA25O	Beverage with sucrose stored at 25 °C under darkness conditions

**Table 2:** pH, titratable acidity (TA), and total soluble solids (TSS) values, measured at day 0 (initial) and after 90 days storage (final), for juices with added stevia or sucrose as sweeteners and stored under three different conditions.

Condition ^Z^	pH			TA (g CA/100 mL)			TSS (°Brix)		
	Initial	Final	*p*-Value	Initial	Final	*p*-Value	Initial	Final	*p*-Value
ST4	3.26	3.16	** ^X^	4.28	4.16	*	7.6	7.7	**
SA4	3.28	3.27	N.s.	3.36	3.34	N.s.	13.7	13.6	N.s.
*p*-Value	N.s.	**		***	**		***	***	
ST25L	3.26	3.21	*	4.28	3.86	**	7.6	7.7	**
SA25L	3.28	3.22	*	3.36	3.61	**	13.7	14.3	*
*p*-Value	N.s.	N.s.		***	*		***	***	
ST25O	3.26	3.25	N.s.	4.28	3.54	***	7.6	7.6	N.s.
SA25O	3.28	3.23	*	3.36	3.66	***	13.7	14.3	*
*p*-Value	N.s.	N.s.		***	**		***	***	

^Z^ ST4, stevia/4 °C; SA4, sucrose 4 °C; ST25L, stevia/25 °C/under light; SA25L, sucrose 25 °C/under light; ST25O, stevia/25 °C/under darkness; SA25O, sucrose 25 °C/under darkness. Initial and final values were significantly different according to paired *t*-test. ^X^ Significant at * (*p* < 0.05), ** (*p* < 0.01) and *** (*p* < 0.001) according to a paired *t*-test. N.s., no significant differences.

**Table 3:** CIEL*a*b* values in beverages stored at 4 °C.

Parameter	Days	Stevia	Sucrose	*p*-Value
CIEL*	0	17.35 ± 0.02d ^Z^	22.37 ± 0.01c	*** ^X^
	15	15.27 ± 0.11b	20.95 ± 0.01a	***
	30	15.14 ± 0.01b	21.87 ± 0.01b	***
	45	14.76 ± 0.01a	23.23 ± 0.04d	***
	60	15.79 ± 0.04c	25.55 ± 0.01e	***
	90	33.67 ± 0.01e	35.93 ± 0.01f	***
CIEa*	0	47.61 ± 0.14d	48.64 ± 0.03d	***
	15	42.90 ± 0.19c	47.97 ± 0.02c	***
	30	42.32 ± 0.05b	47.74 ± 0.01b	***
	45	41.70 ± 0.01a	44.57 ± 0.03a	***
	60	42.76 ± 0.01c	50.57 ± 0.04e	***
	90	57.32 ± 0.01e	60.09 ± 0.01f	***
CIEb*	0	29.31 ± 0.01e	36.82 ± 0.07c	***
	15	26.31 ± 0.16c	34.90 ± 0.02a	***
	30	25.96 ± 0.04b	36.12 ± 0.05b	***
	45	25.25 ± 0.01a	37.89 ± 0.01d	***
	60	26.90 ± 0.08d	40.66 ± 0.04f	***
	90	45.05 ± 0.01f	38.93 ± 0.01e	***
**Chroma**	0	52.55 ± 0.01d	61.01 ± 0.06d	***
	15	50.32 ± 0.07c	59.32 ± 0.02b	***
	30	49.65 ± 0.06b	59.86 ± 0.02c	***
	45	48.76 ± 0.01a	58.50 ± 0.01a	***
	60	50.52 ± 0.09c	64.89 ± 0.01e	***
	90	72.90 ± 0.01e	71.60 ± 0.01f	***
**Hue angle**	0	33.91 ± 0.01c	37.13 ± 2.41c	***
	15	31.52 ± 0.27a	36.04 ± 0.01b	**
	30	31.53 ± 0.01a	37.11 ± 0.04c	***
	45	31.20 ± 0.02a	40.37 ± 0.03e	***
	60	32.17 ± 0.04b	38.80 ± 0.04d	***
	90	38.17 ± 0.01d	32.94 ± 0.01a	***
**ΔE**	0	0.00a	0.00a	N.s.
	15	5.96 ± 0.03c	2.48 ± 0.01c	***
	30	6.64 ± 0.06d	1.25 ± 0.03b	***
	45	7.62 ± 0.01e	4.35 ± 0.09d	***
	60	5.64 ± 0.08b	5.34 ± 0.01e	*
	90	24.66 ± 0.01f	17.88 ± 0.01f	***

^Z^ Means (*n* = 3) within a column followed by the different lowercase letters (comparison between storage timepoints) are significantly different at *p* < 0.001. Within a row (sweetener comparison), values were comparing using a *t*-test. ^X^ Significant differences at * (*p* < 0.05), ** (*p* < 0.01), and *** (*p* < 0.001). N.s., no significant differences. CIEL*, lightness; CIEa*, redness; CIEb*, yellowness; ΔE, difference or distance between two colors.

**Table 4:** CIEL*a*b* values in beverages stored at 25 °C under light condition.

Parameter	Days	Stevia	Sucrose	*p*-Value
CIEL*	0	17.35 ± 0.02a ^Z^	22.37 ± 0.01b	*** ^X^
	15	21.51 ± 1.09b	21.60 ± 0.01a	N.s.
	30	21.84 ± 0.03bc	24.55 ± 0.01c	***
	45	20.90 ± 0.01b	25.82 ± 0.01d	***
	60	23.48 ± 0.01c	30.56 ± 0.01e	N.s.
	90	32.70 ± 0.01d	33.02 ± 0.01f	***
CIEa*	0	47.61 ± 0.01b	48.64 ± 0.03e	***
	15	46.65 ± 0.91c	46.46 ± 0.01d	N.s.
	30	42.92 ± 0.06b	45.65 ± 0.01c	***
	45	42.63 ± 0.01b	49.26 ± 0.03f	***
	60	39.28 ± 0.01a	43.13 ± 0.01b	***
	90	37.86 ± 0.01a	38.26 ± 0.01a	**
CIEb*	0	29.32 ± 0.01a	36.82 ± 0.01b	***
	15	35.32 ± 1.31b	35.49 ± 0.01a	N.s.
	30	35.87± 0.08b	39.43 ± 0.01c	***
	45	34.58 ± 0.06b	39.88 ± 0.04d	***
	60	38.49 ± 0.05c	45.96 ± 0.01e	***
	90	49.72 ± 0.01d	50.30 ± 0.01f	***
**Chroma**	0	52.55 ± 0.01a	61.01 ± 0.06c	***
	15	58.51 ± 1.52c	58.46 ± 0.01a	N.s.
	30	55.93 ± 0.09b	60.32 ± 0.01b	***
	45	54.89 ± 0.04ab	63.38 ± 0.01f	***
	60	55.00 ± 0.02ab	63.02 ± 0.01d	***
	90	62.49 ± 0.02d	63.19 ± 0.01e	***
**Hue angle**	0	33.91 ± 0.01a	37.13 ± 0.03a	***
	15	37.13 ± 0.48b	37.38 ± 0.01b	N.s.
	30	39.89 ± 0.04d	40.82 ± 0.01d	***
	45	39.05 ± 0.05c	39.00 ± 0.05c	N.s.
	60	44.42 ± 0.04e	46.82 ± 0.01e	***
	90	52.71 ± 0.01f	52.74 ± 0.01f	N.s.
**ΔE**	0	0.00a	0.00a	N.s.
	15	7.81 ± 0.06b	2.67 ± 0.01d	***
	30	9.23 ± 0.02d	4.53 ± 0.01c	***
	45	8.08 ± 0.04c	4.66 ± 0.04b	***
	60	13.83 ± 0.04e	13.46 ± 0.01e	**
	90	27.34 ± 0.01f	20.10 ± 0.01f	***

^Z^ Means (*n* = 3) within a column followed by the different lowercase letters (comparison between storage timepoints) are significantly different at *p* < 0.001. Within a row (sweetener comparison), values were comparing using a *t*-test. ^X^ Significant differences at * (*p* < 0.05), ** (*p* < 0.01) and *** (*p* < 0.001). N.s., no significant differences.

**Table 5:** CIEL*a*b* values in beverages stored at 25 °C under dark condition.

Parameter	Days	Stevia	Sucrose	*p*-Value
CIEL*	0	17.35 ± 0.02a ^Z^	22.37 ± 0.01a	*** ^X^
	15	20.83 ± 0.27c	24.60 ± 0.01d	**
	30	19.94 ± 0.02b	23.70 ± 0.01c	***
	45	23.68 ± 0.01d	22.62 ± 0.03b	***
	60	22.25 ± 0.01e	29.64 ± 0.01e	***
	90	35.14 ± 0.01f	34.60 ± 0.01f	***
CIEa*	0	47.61 ± 0.01c	48.64 ± 0.03f	***
	15	46.45 ± 0.18e	47.47 ± 0.03e	*
	30	44.66 ± 0.04d	45.93 ± 0.02c	***
	45	39.85 ± 0.01a	46.99 ± 0.06d	***
	60	40.08 ± 0.01a	40.27 ± 0.01a	***
	90	41.36 ± 0.01b	40.90 ± 0.02b	**
CIEb*	0	29.32 ± 0.01a	36.82 ± 0.07a	***
	15	34.59 ± 0.35c	39.69 ± 0.03c	**
	30	34.43 ± 0.09b	38.21 ± 0.01b	***
	45	38.67 ± 0.01e	37.05 ± 0.13a	**
	60	36.80 ± 0.07d	46.18 ± 0.06d	***
	90	50.13 ± 0.14f	49.24 ± 0.04e	**
**Chroma**	0	52.55 ± 0.01a	61.01 ± 0.06b	***
	15	57.92 ± 0.35d	61.88 ± 0.04c	**
	30	55.79 ± 0.09c	59.74 ± 0.03a	***
	45	55.53 ± 0.01c	59.84 ± 0.13a	***
	60	54.42 ± 0.05b	61.26 ± 0.06b	***
	90	64.99 ± 0.11e	64.01 ± 0.04d	**
**Hue angle**	0	33.91 ± 0.01a	37.13 ± 0.04a	***
	15	36.67 ± 0.17b	39.90 ± 0.01d	**
	30	36.81 ± 0.04b	39.76 ± 0.01c	***
	45	44.14 ± 0.01d	38.26 ± 0.06b	***
	60	42.56 ± 0.06c	48.92 ± 0.04e	***
	90	50.48 ± 0.06e	50.29 ± 0.01f	N.s.
**ΔE**	0	0.00a	0.00a	N.s
	15	6.44 ± 0.39c	3.82 ± 0.01d	*
	30	5.69 ± 0.04b	3.33 ± 0.01c	***
	45	13.71 ± 0.01e	2.21 ± 0.01b	***
	60	11.70 ± 0.05d	14.51 ± 0.03e	***
	90	28.09 ± 0.09f	19.07 ± 0.01f	***

^Z^ Means (*n* = 3) within a column followed by the different lowercase letters (comparison between storage time-points) are significantly different at *p* < 0.001. Within a row (sweetener comparison), values were comparing using a *t*-test. ^X^ Significant differences at * (*p* < 0.05), ** (*p* < 0.01) and *** (*p* < 0.001). N.s., no significant differences.
